# Applying early health technology assessment (e-HTA) to inform investment in novel health technologies in the US

**DOI:** 10.1017/S0266462325100275

**Published:** 2025-07-08

**Authors:** Zizi Elsisi, William Canestaro, L. Steuten, Ryan Hansen

**Affiliations:** 1 The Comparative Health Outcomes, Policy, and Economics (CHOICE) Institute, School of Pharmacy, University of Washington, Seattle, WA, USA; 2 The Foster School of Business, University of Washington, Seattle, WA, USA; 3 Washington Research Foundation, Seattle, WA, USA; 4 Office of Health Economics, London, UK

**Keywords:** health technology assessment, cost–benefit analysis, economic evaluations, innovation adoption, biomedical technology

## Abstract

**Objectives:**

Evaluate how a foundation-supported fellowship employs early health-technology assessment (eHTA) to guide the development and positioning of emerging health innovations.

**Methods:**

We reviewed all eHTA reports conducted under the Fellowship from 2018 to 2021 (*n* = 10), extracting technology class, development stage, economic modeling, and recommendations. In 2023, we conducted thirty-minute structured video interviews with developers of each technology (eleven invitees, ten responses). The interview comprised Likert questions on perceived usefulness and intention to update the model in later stages, and six open-ended questions on perceived advantages, implementation barriers, and downstream actions. Likert data were summarized descriptively; open-ended responses were summarized and discussed within the research team until consensus on key themes.

**Results:**

The eHTA subject technologies were four diagnostics, three therapeutics, two predictive algorithms, and one curative device, all preclinical. Analyses used six Markov or decision-tree frameworks, four hybrid models or simulations, and six value-based-pricing scenarios. Five technologies were potentially cost-effective, three conditionally cost-effective, one unlikely to be cost-effective without stronger evidence, and one cost-effective yet unlikely to break even. Eight developers rated eHTA “useful” or “very useful”; three had already leveraged results in grant or investor materials and two planned to do so when more data emerged. Reported barriers included evidence gaps, funding constraints, and misalignment with pharmaceutical partners on codevelopment strategies; two projects were discontinued.

**Conclusions:**

eHTA supplies developers with early economic insight, but its guidance is most reliable when interpreted alongside budget impact, feasibility, regulatory, and adoption considerations.

## Introduction

The healthcare landscape is undergoing rapid change as advanced technologies move from laboratories into clinical practice. These innovations have the potential to reshape patient care, broaden therapeutic options, reduce health disparities, and streamline service delivery. Yet only a fraction ultimately achieves that promise (1–5); many never clear the economic and implementation hurdles that stand between early-stage proof‑of‑concept and widespread adoption, especially in resource-limited settings where financial constraints and equity concerns are most acute (6). Early assessment of technologies for their potential clinical, economic, and societal value can help to inform decisions about whether, and if so, how, to invest in their further development.

There is a growing interest in early health technology assessment (eHTA). eHTA gathers evidence from initial bench and animal studies, as well as throughout the technology’s translational research phases (7). One proposed definition of eHTA by IJzerman et al. is “all methods used to inform industry and other stakeholders about the potential value of new medical technologies in development, including methods to quantify and manage uncertainty” (8). The broad aim is to offer guidance to technology developers, manufacturers, and investors regarding potential safety, effectiveness, cost-effectiveness, or other economic impacts, and other potentially important impacts, as appropriate. Such analyses aid stakeholders in anticipating the potential commercial feasibility of their technology and provide enhanced direction for technology positioning, potential utilization, and estimated reasonable market pricing (7;8).

Various quantitative approaches have been suggested for use in eHTAs, such as real options analysis, value of information analysis, multicriteria decision analysis (MCDA), clinical trial simulation, and early health economic modeling, among others (8;9). The majority of published eHTAs utilize early health economic modeling approaches such as cost-effectiveness and cost-utility analyses from the perspectives of various stakeholders, including healthcare payers and systems as well as society at large. These models can also be used for headroom estimates, exploring threshold prices for the new technology at various willingness-to-pay thresholds, and identifying values of critical parameters such as clinical effectiveness or diagnostic test performance. In order to address uncertainty, these analyses commonly incorporate deterministic or probabilistic sensitivity analysis, as well as robust scenario analyses to explore the implications of critical assumptions in technology development scenarios (8;10;11).

Despite the growing utilization of eHTAs, there remains a necessity to understand the practical implications of these assessments within real-world scenarios. It is essential to ascertain whether manufacturers and other technology developers find utility in some form of eHTA and, if they do, to explore the specific ways in which eHTA has yielded benefits once a technology has been adopted. We sought to explore whether the results of eHTA and related economic modeling influenced further development and positioning of novel health technologies. We focus on the economic aspects of eHTA, limited to cost-effectiveness analysis (CEA), cost-utility analysis (CUA), budget impact (BI), and return on investment (ROI) models, as suggested by IJzerman et al. and Gutters et al. (7;10). We also use the terminology “early health technology assessment,” “early health technology evaluation,” and “early health economic modeling” synonymously.

## Methods

We performed a descriptive review of all of the eHTAs that were conducted at the University of Washington (UW) and supported by the private, nonprofit Washington Research Foundation (WRF) from 2018 to 2021. The WRF is a private, nonprofit biomedical-research funder and early-stage investor in Washington State. It awards institutional grants to de-risk promising academic technologies and, through its investment arm, WRF Capital, provides early-equity financing to biotech start-ups that license UW intellectual property (12). Since 2018, the WRF has funded a Health Economics and Outcomes Research (HEOR) Fellowship at UW’s CHOICE Institute. Each year, one HEOR trainee, typically a PhD or master’s student in health economics and policy, partners with a Washington-based technology developer to conduct an eHTA. Under a UW School of Pharmacy mentor and WRF representative, the fellow and developer define the product, clinical context, and evidence needs before jointly selecting the analytic approach. In one case, clinical evidence gaps prompted a systematic literature review prior to model construction; all other projects moved directly to proposal drafting, economic modeling, and final report delivery. This flexible process ensures models are grounded in the best available data. Between 2018 and 2021, the fellowship accepted one trainee annually (from two to three applicants), based on prior economic-modeling coursework and demonstrated eHTA ability. The fellow for 2022 and 2023 was the first author of this manuscript (ZE) (13).

The descriptive analysis of the recently conducted WRF eHTAs involved characterizing the type of technology assessed (therapeutic, diagnostic, medical device, or predictive algorithm), target disease area, stage of development at the time of analysis, the analyses performed, the type of economic modeling used, and recommendations made to technology developers.

The first author contacted all developers whose technologies were assessed during this period, inviting them to thirty‑minute structured online face-to-face interviews to discuss their evaluation experience and provide an update on their technology’s status in 2023. These structured interviews involved using a discussion guide of a predetermined set of nine questions, as detailed in Supplementary file. Among these questions, three employed a five-point Likert scale. Two of these questions gauged the overall usefulness of the eHTAs and their effectiveness in guiding technology positioning and further development. The available response options were “very useful,” “useful,” “neutral,” “not useful,” and “absolutely not useful.” The third Likert scale question inquired about the likelihood of technology developers revising their analysis once the technology reached full development, with response options being “very likely,” “likely,” “neutral,” “unlikely,” and “very unlikely.” The remaining six questions were open-ended and aimed to understand the perceived advantages and challenges associated with the assessment process, as well as how the obtained results influenced the technology development efforts. The interviews were recorded with interviewees’ permission and transcribed. We used Microsoft Excel® for the descriptive analysis of our eHTAs database as well as analyzing responses to the Likert scale questions in the interview. For the open-ended questions, the interviewer (ZE) summarized the responses and reviewed these with coauthors to gain their views on emergent themes.

## Results

### Descriptive analysis

Between 2018 and 2021, the period during which these eHTAs were conducted, we evaluated ten early-stage health technologies (see Table 1). Many of these technologies were novel diagnostic devices, accounting for four of the assessed innovations, followed by three therapeutic drugs, two predictive algorithms, and one curative medical device. At the time of each eHTA, all ten innovations were in the preclinical stage, indicating that our economic evaluations did not rely on clinical trial data. (By the time of the interviews, one technology had since advanced into early clinical trials.)

**Table 1.** Overview of the early health technology assessments (eHTAs)

eHTA	Type of technology	Target disease area	Type of analyses	Type of economic model (if any)	Goal of the eHTA	Findings
1	Therapeutic drug	Idiopathic pulmonary fibrosis	Economic evaluation (CUA)	Discrete event simulation	1. Explore potential cost-effectiveness of available therapies in the market. 2. Explore potential cost-effectiveness of new therapy at different efficacy levels 3. Determine VBP and drivers of value	1. Available therapies in the market are not cost effective by commonly used thresholds 2. VBP largely depends on the average length of treatment 3. A new therapeutic could be of high value
2	Therapeutic drug	Hepatitis B	Economic evaluation (CUA)	Markov state transition model	Explore potential cost-effectiveness of new drug under different scenarios	Hypothetical drug is cost saving and is cost-effective in scenarios where it is an add-on therapy
3	Prediction algorithm	Glioblastoma multiforme	Decision analytic model	Decision tree	Estimate the return on investment associated with traditional drug development process vs using the novel prediction algorithm	The novel prediction algorithm can improve the overall probability of identifying an effective therapy combination for glioblastoma multiforme, resulting in higher chances of retrieving R&D costs
4	Diagnostic device	Hypogonadism	Systematic review and economic evaluation (CUA)	Hybrid (Decision tree and Markov model)	1. Evaluate whether there is a difference in efficacy and adverse event due to different race profiles in available therapy 2. Explore the potential cost-effectiveness of available therapy 3. Determine added value of diagnostic device	Investing in the technology is not recommended (potentially not cost-effective)
5	Curative medical device	Intra-abdominal and subcutaneous abscess	Economic evaluation (CEA) and budget impact	Decision tree	Explore the potential cost-effectiveness and breakeven time for two indications in different settings. Breakeven time was defined as the first year where reimbursement exceeded the full cost of care with the device, including capital purchase and maintenance fees.	The device is cost-effective in both indications. However, the device might not be breakeven in one of the indications.
6	Prediction algorithm	Sepsis	Economic evaluation (CUA)	Hybrid (Decision tree and Markov model)	Explore the potential cost-effectiveness of using the prediction algorithm in sepsis risk-stratification technologies	Very likely to be cost-effective
7	Therapeutic drug	Crohn’s disease	Economic evaluation (CUA)	Markov state transition model	Determine VBP of novel therapy in different efficacy scenarios	VBP decreases as the efficacy of novel therapy increases. This is driven primarily by the fact that the cost of the active disease state is much lower than the potential cost of treatment.
8	Diagnostic device	Peanut allergy	Economic evaluation (CEA)	Decision tree	Explore the potential cost-effectiveness and determine VBP of novel device	Cost-effectiveness depends on the diagnostic characteristics and set price
9	Diagnostic device	Biopsy	Economic evaluation (CEA)	Markov state transition model	Explore the potential cost-effectiveness of device in different tissue types	Likely cost-effective in certain tissues
10	Diagnostic device (14)	Bacterial Vaginosis	Economic evaluation (CUA)	State-transition microsimulation model	Explore the potential cost-effectiveness of the novel device	Likely cost-effective from a healthcare perspective and cost-saving from a societal perspective

(1 4): Jiao B, Fredricks DN, Srinivasan S, Hansen R. Economic Evaluation of a Point-of-Care Test for Bacterial Vaginosis Among Women With Vaginal Symptoms. *Sex Transm Dis.* 2023 May 1;50(5):310–6.

Abbreviations: CUA, Cost-utility analysis; VBP, value-based pricing; CEA, cost-effectiveness analysis.

The primary objective of all of the eHTAs was to inform a choice of clinical indication or a focus area among several technically feasible development options, by determining the potential value of the new technologies when compared to existing standard care practices. The primary objective of one eHTA was to calculate the return on investment (ROI) for the novel technology to the manufacturer. A majority of the eHTAs (*n* = six) aimed to estimate the value-based pricing of the novel technologies. In pursuit of these objectives, most of the eHTAs utilized economic evaluations involving multiple sensitivity analyses and use cases of specific subgroups to identify potential value drivers of the innovations. One eHTA included a systematic literature review along with an economic evaluation; another included a budget impact (BI) model, along with a cost-effectiveness analysis, in order to estimate the short-term affordability of that technology.

Six of the eHTAs were categorized as cost-utility analyses (CUA), focusing on determining the incremental cost-utility ratio (ICUR). The remaining eHTAs fell into the category of cost-effectiveness analyses (CEA), where the outcomes were tied to the specific units relevant to the targeted disease area. The economic models primarily utilized three Markov state transition models and three decision trees, along with two hybrid Markov-decision trees, one microsimulation, and one discrete event simulation.

Five of the eHTAs indicated that the innovation had the potential to be cost-effective or yield a sufficiently high ROI, given its intended use. In another three cases, the eHTAs suggested that the technology could become cost-effective under specific circumstances, for instance, if used for a different indication. In one eHTA, further investment in the technology was not encouraged because of potential cost‑effectiveness concerns. However, this conclusion stemmed largely from insufficient data on the device’s efficacy across racially diverse patient groups, and additional evidence could reveal population subgroups in which the device may be cost‑effective. Finally, one eHTA incorporated a cost-effectiveness analysis and a BI model. In that instance, the technology was cost-effective; however, the BI analysis, which estimated the cumulative expected margin over the device’s useful life and the time to break even across hospital segments, showed that although the innovation would break even in one indication, it might still fail to do so for developers and their investors in the other indication. All of these recommendations were communicated directly to the developers in the fellow’s final report.

### Follow-up interviews

**Figure 1.**
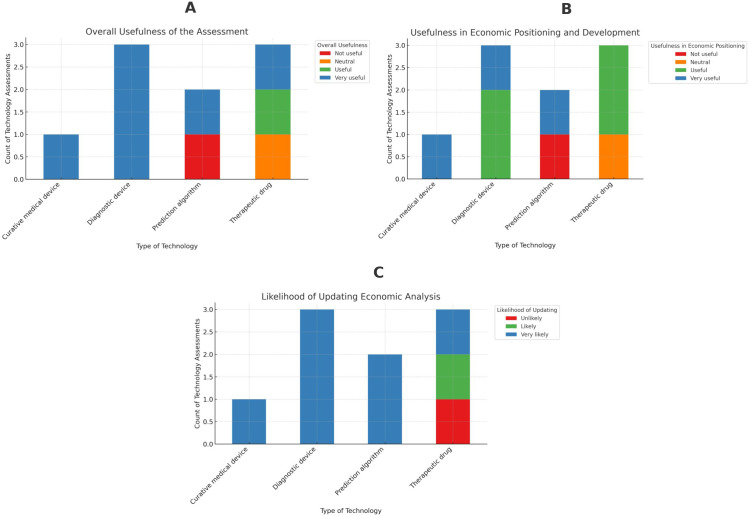
A summary of the Likert-scale questions grouped by the type of technology. Panel A illustrates the responses of the interviewees to the question of the overall usefulness of the assessment. Panel B shows the responses to the usefulness of the assessment in economic positioning and development of the technology. Panel C represents the responses to the likelihood of updating the economic analysis.

We invited eleven of the technology developers, including two who were responsible for the same technology, for a thirty-minute structured interview; ten agreed to be interviewed. Detailed interview results for each eHTA are shown in Supplementary Table 1. When interviewees were asked to evaluate the utility of the economic analysis and technology projections, seven rated it as “very useful,” one as “useful,” one as “neutral,” and one as “not useful.” All technology developers agreed the analysis improved their understanding of market dynamics and economic implications, with several noting it boosted confidence in their technology’s potential market value. Aspects of market dynamics that they cited included, for example, clarifying the prospective target population, identifying likely competitors, estimating a value‑based price corridor, pinpointing evidence gaps, and, when multiple indications were possible, determining which indication offered the safest initial investment. Interviewees’ utility for the eHTAs varied by technology type. All diagnostic device developers rated it “very useful,” although one-third of therapeutic drug developers rated it “not useful.” Figure 1 illustrates these findings. Despite this, eight rated the analysis as “very useful” or “useful” in terms of economic positioning and technology development, with two of the three therapeutic drug developers affirming its value.

Of the eight developers who found the analysis useful, only two incorporated it into grant applications, and one shared the findings with potential investors. Two developers had not yet utilized the analysis but planned to do so, citing the need for additional data or funding. One developer who rated the analysis as “very useful” noted current barriers to technology adoption but highlighted its potential for future investments. Conversely, a developer who rated the analysis “not useful” still used it in discussions with the pharmaceutical industry.

Sensitivity analyses were valuable to half of the developers, helping them define price ranges and confirm findings. At the time of the interview, most technologies were in the preclinical phase, except for one in early clinical trials and others undergoing repurposing. Two technologies of the nine preclinical technologies were discontinued due to challenges in real-world adaptation and a lack of funding.

When asked if eHTA altered their development decisions, six indicated no but acknowledged it provided valuable business and economic insights. One developer identified a second indication for their technology based on the eHTA, which was then incorporated into a grant proposal. Lastly, all developers except one therapeutic drug developer expressed a strong likelihood of updating the analysis at a later-stage HTA, emphasizing the importance of adapting to evolving market conditions. The drug developer would consider updating the economic model if the competitive market changes.

Aligned with the eHTA outcomes (Table 2), therapeutic drug developers were uniformly recommended to pursue their technologies, with two-thirds securing early-stage grant funding and leveraging the analysis in value propositions with investors. Similarly, the two prediction algorithm developers were also recommended to continue, but they faced substantial barriers to real-world adoption and reported the lowest scores on Likert-scale questions regarding the eHTA’s usefulness. Conversely, developers of diagnostic devices who received a “not potentially cost-effective” are gathering additional clinical trial data and view the eHTA as highly useful, with strong intentions to update their models. Similarly, developers who were advised to repurpose their technologies indicated that they adapted their focus while recognizing the eHTA’s critical value in guiding their strategic decisions.

**Table 2.** Summary of the type of technology and the interview results according to the recommendation made to the developer

Recommendation to developer		Type of technology (N)	Overall usefulness	Usefulness of economic positioning and development	Likelihood of updating the economic evaluation	The Use of the eHTA by developer
Potential to be cost-effective		Therapeutic drug (3)				Early-stage grant funding; Value proposition with investors; development of technology is halted
		Diagnostic device (1)				Grant funding and analysis published
		Prediction algorithm (2)				Real-world barriers to technology adoption
No potential to be cost-effective		Diagnostic device (1)				Model will be used for larger data sets collected from early clinical phase trials
Potentially cost-effective in certain settings/ indications		Diagnostic device (1)				Repurposing to certain recommended tissue type
		Curative device (1)				Grant funding

Response gradient:

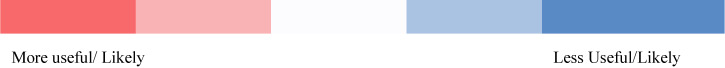

## Discussion

The results of this study show the usefulness of eHTA in shaping the development and commercialization of health technologies within the United States. Our findings reveal four main insights. First, early health technology assessments and early health economic modeling can help to distinguish between novel technologies that hold the potential for being cost-effective for a primary indication, those that may only be cost-effective under specific circumstances (such as particular indications and settings), and those that may not prove cost-effective unless further evidence can be gathered. This differentiation is instrumental in providing guidance to manufacturers and technology developers, helping them make informed decisions about resource allocation and whether to proceed with the development of a particular technology or explore alternative paths.

Second, the analysis underscores that eHTA extends beyond the metric of cost-effectiveness of technologies. For instance, six of these eHTAs examined value-based pricing. In one interview, technology developers emphasized the significance of comprehending the pricing dynamics of their technologies and how these dynamics might be influenced by the specific attributes of their diagnostic devices. Third, our interviews demonstrate a generally positive reception of eHTA: most developers rated eHTA results as “useful” or “very useful” and said they would revisit the analysis as their technology matured, using the outputs in grant proposals and investor discussions. However, few interviewees expressed that eHTA offered limited practical value. One developer of a combination drug argued that deeper, earlier engagement with pharmaceutical partners would matter far more than early economic signals, because that form of therapy was intrinsically ill-suited to the current typical pipeline, where each component drug is manufactured by a different company and already approved for separate indications. In their view, these structural hurdles, not cost‑effectiveness metrics, ultimately dictate go/no‑go decisions.

Finally, it is essential to recognize that early indications that a technology is potentially cost-effective do not automatically ensure its commercial viability. The findings from both the descriptive analysis and the interviews revealed instances where certain technologies were deemed potentially cost-effective or showed promising ROI. However, these technologies faced practical hurdles when it came to continued development and real-world implementation. There were also cases where the eHTA suggested that a technology could be cost-effective, but the BI model indicated that the technology might not achieve financial sustainability for a specific indication. This highlights that while early health economic modeling can play a useful role in assessing value and guiding decision-making by manufacturers and technology developers, it is only one facet of a multifaceted landscape. Other factors, such as budget constraints, feasibility, and the broader diffusion of the technology, including regulatory and payment hurdles as well as adoption by clinicians, are equally important considerations in shaping the trajectory of technology investments and realizing their full potential (15;1 6).

A prior study conducted by Grutters et al. assessed thirty-two early economic models encompassing thirty health innovations and reported that each retained at least some potential for cost‑effectiveness (10). Our results align with this general pattern, as none of the ten technologies evaluated in the present study warranted an unequivocal “no‑go” recommendation. Nevertheless, one technology was classified as potentially not cost‑effective because both base‑case and sensitivity analyses indicated that, without substantially stronger efficacy data or a lower price, its incremental cost-effectiveness ratio would exceed accepted willingness‑to‑pay thresholds. Two additional projects were deemed viable only contingent upon major strategic repositioning or further evidence generation. The comparatively more cautious tone of our recommendations may reflect the developmental maturity of the samples, as nine of our ten projects were still in pre‑clinical stages, whereas a larger proportion of the innovations examined by Grutters et al. had progressed to later clinical phases, enabling those authors to populate their models with more robust data and, consequently, to project more confident value profiles. Regardless, our study concluded that these eHTAs served as valuable tools for gaining insights into the scope and the associated uncertainty regarding the potential cost-effectiveness of each technology. Consistent with our own findings, the eHTAs by Grutters et al. also proved that eHTA helps in providing guidance on how to proceed with the further development and positioning of the respective technologies, as well as to direct future research.

To the best of our knowledge, our study represents the first attempt to assess the ramifications of implementing eHTA within a U.S. context. Published reports of eHTAs remain scarce; a literature review by Koufopoulou et al. identified only seven U.S.‑based eHTAs (17). By helping to augment the knowledge base, our analysis is especially timely given the ongoing shifts in U.S. drug‑pricing policy and the potential rise in the use of HTA methods. Nonetheless, it is important to acknowledge that this research has certain limitations. First, we were unable to provide greater detail about the assessed technologies due to their proprietary nature, which is a common challenge encountered in early HTA. Second, although our follow-up interviews yielded valuable insights, they are confined to technology developers operating in the preclinical/early clinical phase. To gain a more comprehensive understanding of how eHTA methods influence technology development, it is imperative to conduct interviews with developers once their technologies have reached full maturity. Finally, our analysis is based on ten eHTAs conducted through a single Washington State‑based program that targets early‑stage innovations emerging from academic laboratories or local start‑ups. Consequently, the technologies and developers included here do not represent the full diversity of early R&D pipelines elsewhere in the United States. In addition, some developers may engage in part because continued collaboration can facilitate access to future WRF grant funding. Such self‑selection could bias the sample toward stakeholders who are more receptive to economic evidence and therefore more likely to view eHTA favorably. Voluntary participation in the follow‑up interviews introduces further risk of non‑response and social‑desirability biases, as those developers who remained committed to their projects may have been more inclined to provide positive feedback. These factors may have led to over-estimating the perceived usefulness of eHTA and developers’ stated intentions to update their models. Future research should aim to encompass a larger sample size to enhance the robustness of the conclusions drawn.

eHTA gives developers an economic lens; it can generate projections of whether a concept is likely to be cost‑effective, map a value‑based price corridor, and explore which indications or care settings could maximize returns. Most developers in our study viewed these analyses as helpful because they clarified market dynamics and the potential economic value of their innovations. Yet, economic analysis can provide only part of the development outlook. Budget impact on payers and providers, technical and operational feasibility, regulatory and reimbursement hurdles, and the prospects for adoption can all override a favorable early cost‑effectiveness signal. eHTA is therefore most powerful when integrated into a broader framework that weighs these additional factors, yielding a more rounded basis for go/no‑go and positioning decisions.

## Supporting information

Elsisi et al. supplementary material Elsisi et al. supplementary material

## Supplementary material

The supplementary material for this article can be found at http://doi.org/10.1017/S0266462325100275.
