# Supplementary material for: Applying early health technology assessment (e-HTA) to inform investment in novel health technologies in the US
Source: Int J Technol Assess Health Care. 2025 Jul 8;41(1):e45. doi: 10.1017/S0266462325100275 (PMC12303690; doi:10.1017/S0266462325100275)
Supplement: Elsisi et al. supplementary material [file S0266462325100275sup001.docx]

**Appendix 1:**

**Interview questions**

1. How useful was the information that you took away from the fellow’s economic analysis and projections about your technology?
2. Very Useful
3. Useful
4. Neutral
5. Not Useful
6. Absolutely Not Useful
7. What do you think was the most useful parts of the fellowship?
8. What do you think was the most challenging part of the fellowship?
9. How useful were the results of the economic analysis in positioning and further developing your technology?
10. Very Useful
11. Useful
12. Neutral
13. Not Useful
14. Absolutely Not Useful
15. How did you apply the results of the economic analysis in positioning and further developing your technology?
16. How useful were the specific sensitivity analyses performed by the fellow to your product development decision?
17. Could you describe what stage of development your technology was at during the time of the HEOR fellowship. Where is it now?
18. If you had not gone through the experience of working with a WRF HEOR Fellow, what different decision would you have taken with regard to your product development?
19. Would you consider updating the economic analysis after the product has been fully developed and ready to enter the market? (later stage HTA)
20. Very Likely
21. Likely
22. Neutral
23. Unlikely
24. Very Unlikely

**Table 1: Results of the Follow-up interviews**

| **Assessment** | **Overall usefulness of the assessment (Q1)** | **Feedback about analysis (Q2 and Q3)** | **Usefulness in economic positioning and development of product (Q4)** | **Applying results of economic analysis (Q5 and Q6)** | **Stage of technology during assessment and in 2023 (Q7)** | **Different decision if no eHTA (Q8)** | **Likelihood of updating economic analysis (Q9)** |
| --- | --- | --- | --- | --- | --- | --- | --- |
| 1 | Very useful | Most useful: Validation of the need to develop a new therapeutic drug in the idiopathic pulmonary fibrosis space  Most challenging: understanding the economic analysis and underlying assumptions | Useful | - Results of the analysis have been used in conversations with investors about value propositions  - Usefulness of sensitivity analyses: was not of much use | Pre-clinical phase | Decision would have not been different. The developer also used a different form of analysis that showed the innovation is valuable | Unlikely (unless the competitive marketplace is changed) |
| 2 | Neutral | Most useful: Identifying and understanding the different potential markets for the product  Most challenging: analysis depends on many assumptions, making it difficult to predict the reliability of the projections in different markets (USA vs East Asia) | Neutral | - Results of the analysis had not been used yet  - Usefulness of sensitivity analyses: was not used | Pre-clinical phase (development of technology is halted due to failure to allocate funding) | Decision would have not been different | Likely |
| 3 | Not useful | Most useful: better understanding of the calculations and information needed to project the economic impact of the technology  Most challenging: communicating the specific capabilities of the technology for the fellow to execute the right modelling framework | Not useful | - Results of the analysis have been used in conversations with the pharmaceutical industry, but it is hard for the technology to be adapted in real world  - Usefulness of sensitivity analyses: was not used in conversations with the pharmaceutical industry. However, it was useful for the developer to confirm the robustness of the model | The technology is being re-purposed for a new indication and setting | Decision would have not been different, but there would be a missed opportunity for learning about HEOR analysis | Very likely |
| 4 | Very useful | Most useful: great multidisciplinary training opportunity, greater understanding of advanced statistics and outcomes research  Most challenging: limited studies were available to support the concept of the innovation | Useful | - Results of the analysis have not been used yet, but the model will be used as a template for larger data sets in the future  - Usefulness of sensitivity analyses: Neutral | Early clinical trial phase | Decision would have not been different, but there would have been a lack of understanding the business and economic side of the technology | Very likely |
| 5 | Very useful | Most useful: validation of understanding the potential of the technology in the market, and the discovery of a new indication for the technology  Most challenging: defining the research question for the economic study | Very useful | - Results of the analysis have not been used yet, but developers intend to use it for government grant funding, as well as requesting funds from banks and venture capitals  - Usefulness of sensitivity analyses: Confirmation that the analysis is robust, and technology can be commercialized | Pre-clinical phase | No realization of the possibility of a second indication and would not have received grant funding for that indication | Very likely |
| 6 | Very useful | Most useful: getting acquainted with HEOR methods, realizing that this work is feasible within graduate student level resources  Most challenging: deciding which project to choose for this analysis | Very useful | - Results of the analysis have not been used yet, since there are currently barriers to the adoption of the technology. But future investment in the technology could be possible  - Usefulness of sensitivity analyses: useful but need to consider that there were a lot of assumptions | Pre-clinical phase | Investment in the product would have received a lower position in the priority list | Very likely |
| 7 | Useful | Most useful: gaining information on overall market size, and helping in determining target population  Most challenging: No challenges | Useful | - Results of the analysis have been used for early-stage grants and internal education  - Usefulness of sensitivity analyses: not used | Pre-clinical proof of concept phase | Decision would have not been different | Very likely |
| 9 | Very useful | Most useful: can build on the model when the product is more developed and results are clear  Most challenging: time consuming process to develop the model that accurately reflects the research question | Useful | - Results of the analysis were useful in gaining confidence regarding the potential benefit of the technology. However, it was not part of the grant submission  - Usefulness of sensitivity analyses: useful as it is a key component of the model | Early pre-clinical phase. (technology is re-purposed to a certain type of tissue) | Decision would have not been different, but there would be more uncertainty with regards to the economic value of the innovation | Very likely |
| 10 | Very useful | Most useful: understanding technology beyond the perspective of the developer, academic enriching experience, having an objective metric for grant applications and investors  Most challenging: overall no challenges | Very useful | - Results of the analysis have been published and will be used for grant application  - Usefulness of sensitivity analyses: better understanding of acceptable range of prices for the technology, the drivers of cost, and the acceptable sensitivity and specificity metrics to achieve target price | Early clinical trial phase | Less clear on which pathway to take for technology development | Very likely |

Abbreviations: HEOR, Health Economics and Outcomes Research.
